# Glanders

**Published:** 1895-06

**Authors:** 


THE *Journal of Comparative Pathology and Therapeutics*, in a leading editorial, calls for better regulations and laws concerning the existence of glanders, and its more thorough eradication. It demands the adoption of mallein as a test agent in all infected stables and studs, isolation and destruction of all animals responding to the test; advises a strong penalty for selling a glandered horse; makes the purchase price recoverable at law; quarantining of all infected premises until free from the disease demonstrated by the mallein test—all of which is just and right and in the direction of a better condition of affairs and the ultimate suppression of the disease. Fortunately for us in America most of our States have strict laws on the subject, and laws compelling the destruction of all known cases, with severe penalties for trafficking in glandered animals.

